# Supplementary figures and images for: An amino acid substitution in HCV core antigen limits its use as a reliable measure of HCV infection compared with HCV RNA
Source: PLoS One. 2023 Jun 29;18(6):e0287694. doi: 10.1371/journal.pone.0287694 (PMC10310030; doi:10.1371/journal.pone.0287694)

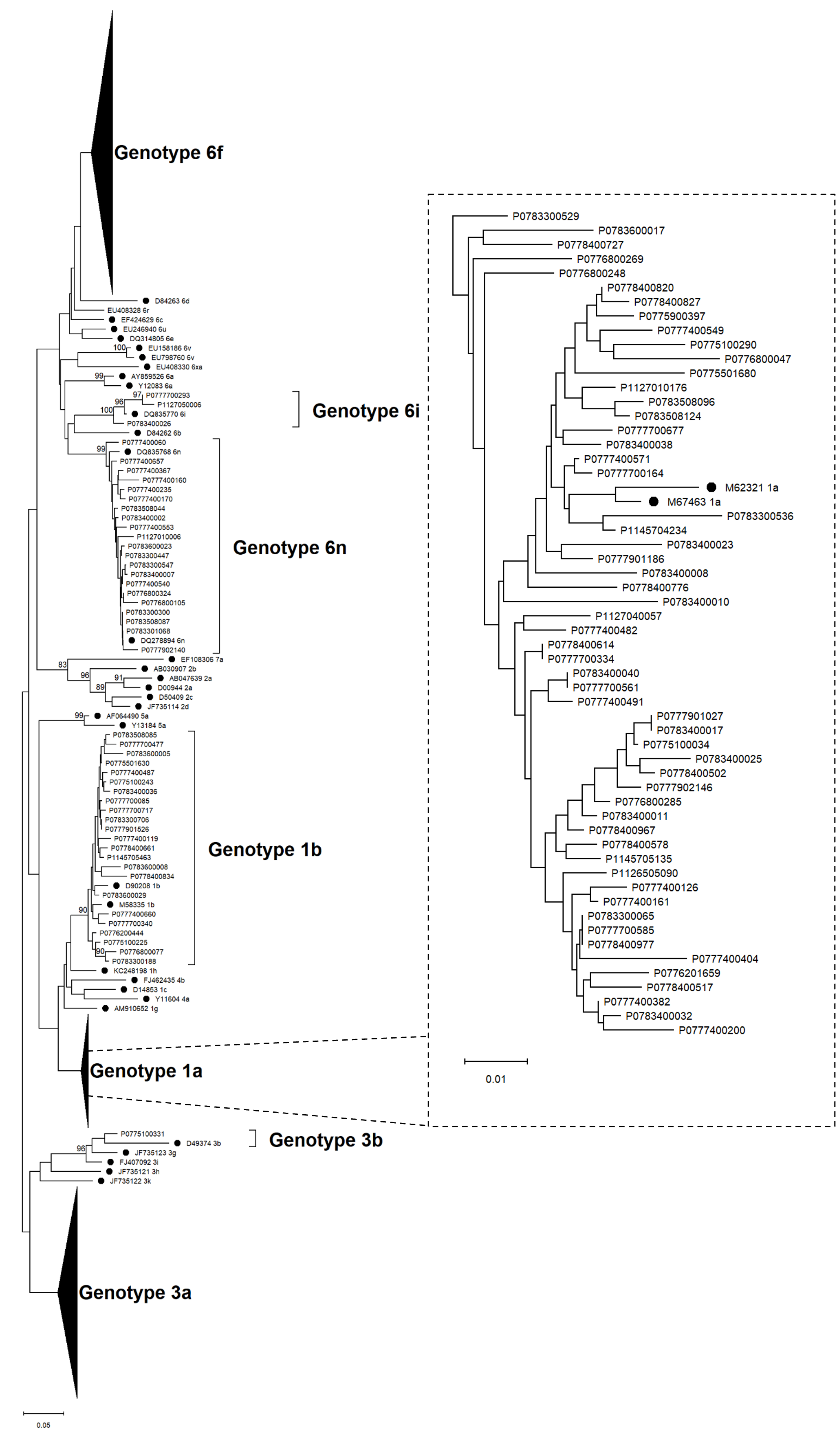

Supplement: S1 Fig — (TIF) [file pone.0287694.s001.tif]

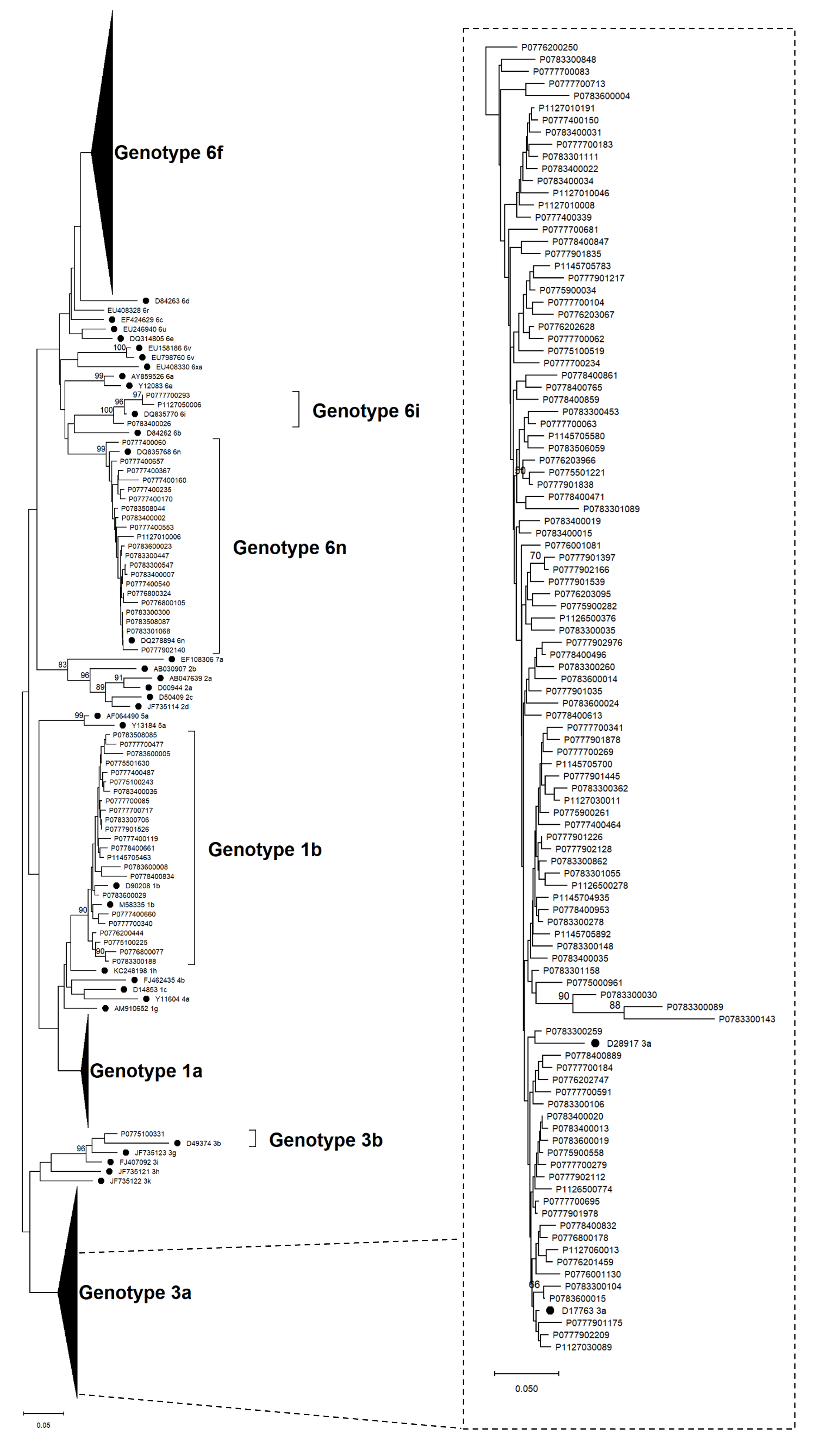

Supplement: S2 Fig — (TIF) [file pone.0287694.s002.tif]

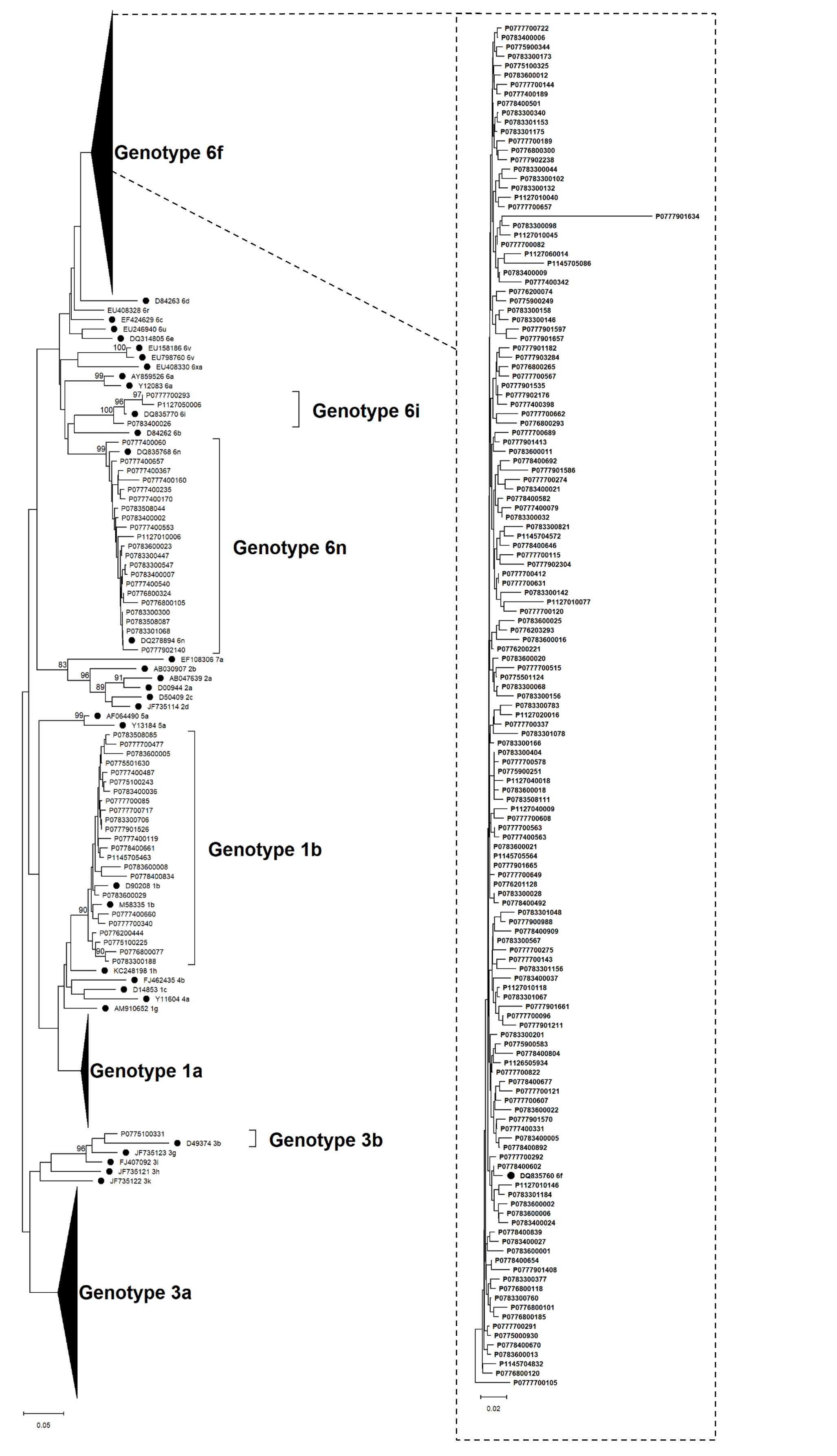

Supplement: S3 Fig — (TIF) [file pone.0287694.s003.tif]
