# Supplementary material for: An amino acid substitution in HCV core antigen limits its use as a reliable measure of HCV infection compared with HCV RNA
Source: PLoS One. 2023 Jun 29;18(6):e0287694. doi: 10.1371/journal.pone.0287694 (PMC10310030; doi:10.1371/journal.pone.0287694)
Supplement: S1 File — The accession number of case (C) and control (N) of HCV core sample. (DOCX) [file pone.0287694.s004.docx]

**List of Core randomized samples**

| Sample name | Sample ID | Accession number |
| --- | --- | --- |
| N-1 | P0777400339 | OQ351376 |
| N-2 | P0777700183 | OQ351407 |
| N-3 | P0777700695 | OQ351440 |
| N-4 | P0777700269 | OQ351411 |
| N-5 | P0777901035 | OQ351447 |
| N-6 | P0777901226 | OQ351453 |
| N-7 | P0777901978 | OQ351471 |
| N-8 | P0777902166 | OQ351476 |
| N-9 | P0777902128 | OQ351473 |
| N-10 | P0778400496 | OQ351485 |
| N-11 | P0777902976 | OQ351481 |
| N-12 | P1145705700 | OQ351639 |
| N-13 | P0783400022 | OQ351578 |
| N-14 | P0783600024 | OQ351618 |
| N-15 | P1127060013 | OQ351628 |
| N-16 | P0777400189 | OQ351372 |
| N-17 | P0777400540 | OQ351386 |
| N-18 | P0777700096 | OQ351398 |
| N-19 | P0777700121 | OQ351403 |
| N-20 | P0777700337 | OQ351419 |
| N-21 | P0777700649 | OQ351434 |
| N-22 | P0777700657 | OQ351435 |
| N-23 | P0777700689 | OQ351439 |
| N-24 | P0777901182 | OQ351449 |
| N-25 | P0777902238 | OQ351479 |
| N-26 | P0778400582 | OQ351490 |
| N-27 | P1145704832 | OQ351632 |
| C-1 | P0775000961 | OQ351363 |
| C-2 | P0777700234 | OQ351410 |
| C-3 | P0777700341 | OQ351421 |
| C-4 | P0777700713 | OQ351441 |
| C-5 | P0778400889 | OQ351512 |
| C-6 | P0783300148 | OQ351532 |
| C-7 | P0783600004 | OQ351601 |
| C-8 | P0783400037 | OQ351589 |
| C-9 | P1127060014 | OQ351629 |
| C-10 | P1145705086 | OQ351634 |
| C-11 | P0777700144 | OQ351405 |
| C-12 | P0777902140 | OQ351474 |
| C-13 | P0783300142 | OQ351529 |
